# Left atrial volume index as an independent determinant of pulmonary hypertension in patients with chronic organic mitral regurgitation

**DOI:** 10.1186/s12872-016-0306-3

**Published:** 2016-06-22

**Authors:** Nithima Ratanasit, Khemajira Karaketklang, Rungroj Krittayaphong

**Affiliations:** Division of Cardiology, Department of Medicine, Siriraj Hospital, Mahidol University, Bangkok, 10700 Thailand; Department of Medicine, Siriraj Hospital, Mahidol University, Bangkok, 10700 Thailand

**Keywords:** Left atrial volume, Mitral regurgitation, Pulmonary hypertension

## Abstract

**Background:**

The common pathophysiological consequences of chronic mitral regurgitation (MR) are left atrial (LA) remodeling/dilatation and pulmonary hypertension (PH). We aimed to study the association between LA volume (LAV) and PH in patients with chronic organic MR.

**Methods:**

We prospectively studied 154 patients (age 55.0 ± 16.4 years, 39.6 % female) with isolated moderate to severe chronic organic MR. Severity of MR was assessed using proximal isovelocity surface area method. LAV was assessed using the area-length biplane method. PH was defined as pulmonary artery systolic pressure > 50 mmHg.

**Results:**

Ruptured chordae and flail leaflets were the most common etiology of MR (53.2 %). Severe MR (effective regurgitant orifice area (EROA) > 40 mm^2^) was described in 123 (79.9 %) patients. Dyspnea, history of heart failure and atrial fibrillation was reported in 37.7 %, 20.1 % and 29.4 % of patients, respectively. Left ventricular (LV) ejection fraction was 68.1 ± 5.9 %. LAV index and EROA were 67.1 (24.7–391.3) ml/m^2^and 60.3 (10.5–250.9) mm^2^, respectively. Age, presence of atrial fibrillation, EROA, LV end-systolic and end-diastolic volume, LV mass index, LAV index and tricuspid annular plane systolic excursion were all factors univariately associated with PH. In multiple logistic regression analysis, age (OR = 1.03, 95 % CI: 1.001-1.06, *p* = 0.04), EROA (OR = 1.02, 95 % CI: 1.003-1.03, *p* = 0.017) and LAV index (OR = 1.01, 95 % CI: 1.002-1.02, *p* = 0.021) were independently associated with PH.

**Conclusions:**

In patients with chronic organic MR, a significant association exists between LAV index and PH. Age, the severity of MR as assessed by EROA, and LAV index are the independent determinants of PH.

## Background

Mitral valve diseases, mitral stenosis and mitral regurgitation (MR), remain a common health problem worldwide. The typical pathophysiological consequences of mitral valve diseases, regardless of the etiologies, are left atrial (LA) dilatation and pulmonary hypertension (PH). Organic MR is a common problem and its prevalence increases with age [1]. Dilatation of LA, LA remodeling, is a compensatory mechanism for the direct effect of chronic volume overload to LA [2]. The adaptation of LA in MR is to prevent pulmonary congestion, maintain normal LA and pulmonary artery pressure, and avoid PH [3]. As the severity of MR progresses, atrial fibrillation (AF) and PH eventually occur [4, 5]. The assessment of LA can reliably be performed using echocardiography. LA volume (LAV) is an accurate cardiovascular risk marker in patients with organic MR in predicting adverse outcomes [6–8]. The presence of PH, a frequent consequence after chronic organic MR, has prognostic implication pre- and post-operatively [5, 9, 10]. Furthermore, recent guidelines emphasize the clinical importance of PH, defined as pulmonary artery systolic pressure > 50 mmHg, in asymptomatic patients with chronic severe MR as an indication for early mitral valve surgery [11, 12]. The aim of the present study was to determine the correlation between LAV and PH in patients with isolated, chronic organic MR.

## Methods

### Study population

The study cohort consisted of consecutive patients who were referred to the echocardiographic laboratory for clinical evaluation of cardiac disease. An electrocardiogram was obtained in all patients on the day of echocardiography. After the results of echocardiography were available, those with the diagnosis of isolated organic MR were prospectively enrolled in the study. Eligible patients were adults over 18 years of age with the diagnosis of chronic organic MR who had effective regurgitant orifice area (EROA) of MR ≥ 20 mm^2^. Patients excluded from the study were those with insignificant MR (EROA < 20 mm^2^), combined mitral valve disease of significant degree, functional/ischemic MR, previous percutaneous balloon mitral valvotomy, associated significant aortic valve disease (moderate to severe degree), prosthetic valve at any position, previous cardiac or valve surgery, left ventricular (LV) systolic dysfunction (LV ejection fraction < 50 %), congenital or pericardial disease, renal dysfunction (serum creatinine > 2 mg/dL), pulmonary or hepatic disease and those with limited or poor-quality echocardiographic study. The patients were included in the study, regardless of symptoms. Symptomatic patients were defined as those in New York Heart Association function classes II – IV. The institutional review board of Siriraj Hospital, Mahidol University (Bangkok, Thailand), approved the study protocol. Informed consent was obtained from all patients.

### Echocardiography

A comprehensive transthoracic echocardiographic examination consisted of two-dimensional, M-mode, Doppler echocardiography and tissue Doppler imaging (TDI). The average of echocardiographic measurements of 3–5 consecutive cardiac cycles was used for the analysis. The severity of MR was quantitatively assessed using proximal isovelocity surface area method and grading according to the recommendation by American Society of Echocardiography [13]. Patients with severe MR were defined as those with EROA ≥ 40 mm^2^ and regurgitant volume ≥ 60 ml. Tricuspid regurgitation velocity (continuous-wave Doppler) was measured to allow the calculation of pulmonary artery systolic pressure, using the simplified Bernoulli equation [14]. Continuous-wave and pulse-wave Doppler spectra of pulmonic regurgitation were obtained for the determination of mean pulmonary artery pressure, pulmonary artery end-diastolic pressure and pulmonary vascular resistance [14–16]. PH was defined as pulmonary artery systolic pressure > 50 mmHg [11, 12]. Regarding the estimation of pulmonary pressures, right atrial pressure was determined by inferior vena cava diameter and the respiratory collapse according to a report from the American Society of Echocardiography [14]. LA diameter was determined by M-mode echocardiography at aortic valve level of parasternal short axis view [17]. LAV was determined using the biplane area-length method and LV mass was calculated as recommended by the American Society of Echocardiography [17]. LAV and LV mass were indexed for body surface area. The LV end-systolic and end-diastolic dimensions and wall thickness were measured using two-dimensional echocardiography. The LV end-systolic and end-diastolic volume and LV ejection fraction were determined using the Modified Simpson’s rule (biplane) [17]. Longitudinal systolic (S_m_’) myocardial velocity, indicating LV systolic function, was measured in apical 4-chamber view using TDI with the sample volume at the medial mitral annulus. The LV diastolic function was evaluated by Doppler echocardiography of transmitral flow and TDI of the mitral annulus. Peak early (E) and late (A) diastolic velocities of mitral inflow and deceleration time of E were measured using pulsed-wave Doppler with the sample volume at the tip of mitral valve. The TDI determination of diastolic function was performed in apical 4-chamber view with the sample volume at the septal aspect of mitral annulus. Longitudinal early (E’) and late (A’) diastolic myocardial velocities were measured. Right ventricular systolic function was evaluated by assessing both the tricuspid annular plane systolic excursion (TAPSE), determined using M-mode echocardiography at lateral tricuspid annulus, and the peak systolic velocity of lateral tricuspid annulus (S_t_’), measured by TDI.

### Statistical analysis

Clinical and echocardiographic data were analyzed using descriptive statistics, including means, standard deviation, number and percentage. The Student’s t-test or Mann Whitney U test were used to compare continuous variables between 2 groups of patients. Comparison of categorical variables was performed using the Chi-square test. Spearman rank correlation was used to relate the LAV index and the magnitude of MR, pulmonary artery systolic pressure in all patients and the subgroups. The Mann Whitney U test was used to compare LAV index in patients with and without AF. The receiver operating characteristic curve (ROC) analysis was performed to determine the cutoff values of LAV index for predicting PH. Univariate binary logistic regression analysis was used to assess the relationship between PH and other variables. Multiple binary logistic regression analysis was performed using PH as the dependent variable. Risk factors or independent variables (age, presence of atrial fibrillation, LV mass, LV end-systolic and end-diastolic volume, LAV index, TAPSE and EROA) were selected on the basis of clinical and statistical significances. The model was fitted by backward stepwise method for variable selection in and out of the model. All *p*-values are reported as 2-tailed, except where otherwise indicated. A *p*-value of ≤ 0.05 was considered statistically significant. Statistical analyses were performed using SPSS statistical package version 18.0.

## Results

A total of 154 patients (age 55.0 ± 16.4 years, 39.6 % female) were enrolled in the study. The majority of patients (62.4 %) were asymptomatic at the time of echocardiography. Among symptomatic patients, 31.2 %, 5.2 % and 1.3 % were in New York Heart Association functional class II, III and IV, respectively. Severe MR and PH were reported in 123 (79.9 %) and 41 (26.6 %) of patients, respectively. Baseline clinical characteristics in all patients and patients grouped according to the presence of PH are shown in Table 1. Patients with PH were older than those without. More patients with PH were symptomatic, taking diuretic and oral anticoagulant, and had history of heart failure. AF was reported in 45 (29.4 %) patients and more common in patients with PH than those without (46.3 % vs. 23.2 %, *p* = 0.005). More patients with PH had the abnormalities on the electrocardiogram (such as LA enlargement and LV hypertrophy by voltage criteria) than those without (41.5 % vs. 21.4 %, *p* = 0.013 for LA enlargement and 63.4 % vs. 38.4 %, *p* = 0.011 for LV hypertrophy).

**Table 1:** Baseline characteristics in all patients with chronic organic mitral regurgitation and the comparisons between patients with and without pulmonary hypertension

Variables	All patients (*N* = 154)	No PH (*N* = 113)	PH (*N* = 41)	*P*-value
Age (years)	55.0 ± 16.4	53.4 ± 16.7	59.3 ± 14.8	0.050
Male gender	93(60.4)	69(61.1)	24(58.5)	0.777
Symptomatic	58(37.7)	34(30.1)	24(58.5)	0.001
BMI (kg/m^2^)	22.8 ± 3.4	22.6 ± 3.4	23.2 ± 3.4	0.362
Hypertension	61(39.6)	43(38.1)	18(43.9)	0.512
Diabetes mellitus	10(6.5)	8(7.1)	2(4.9)	1.0
Dyslipidemia	44(28.6)	33(29.2)	11(26.8)	0.773
Smoking	46(29.9)	36(31.9)	10(24.4)	0.371
History of HF	31(20.1)	16(14.2)	15(36.6)	0.002
Prior stroke/TIA	8(5.2)	7(6.2)	1(2.4)	0.682
Betablocker	46(30.3)	31(27.9)	15(36.6)	0.302
ACEI/ARB	56(36.8)	39(35.1)	17(41.5)	0.473
Anticoagulant	32(21.1)	19(17.1)	13(31.7)	0.050
Diuretics	65(42.8)	38(34.2)	27(65.9)	<0.001

Data are expressed as mean ± standard deviation and number (percentage)

*P*-values are for comparisons between 2 groups

*ACEI* angiotensin converting enzyme inhibitor, *ARB* angiotensin receptor blocker, *BMI* body mass index, *HF* heart failure, *PH* pulmonary hypertension, *TIA* transient ischemic attack

Regarding the etiology of MR, ruptured chordae and flail leaflets were the most common (53.2 %). Mitral valve prolapse without ruptured chordae or flail leaflet, and isolated rheumatic MR were reported in 54 (35.1 %) and 7 (4.5 %) patients, respectively. Pulmonary artery systolic pressure was higher in patients with severe MR than those with non-severe MR (44.9 ± 18.7 vs. 36.3 ± 10.4 mmHg, *p* = 0.001). Table 2 showed echocardiographic parameters of all patients and the comparisons between patients with and without PH. The number of patients with severe MR was significantly higher in the group of patients with PH. Patients with PH had significantly more severe MR as indicated by larger EROA and regurgitant volume, larger LA and LV sizes, more LV hypertrophy, more severe diastolic dysfunction (shorter deceleration time, higher E/A and E/E’), more impaired right ventricular systolic function (lower TAPSE) and more severe tricuspid regurgitation as determined by vena contracta width. Nevertheless, LV ejection fraction, E’, S_m_’ and S_t_’ were not significantly different between patients with and without PH. Table 3 demonstrated that patients with PH had significantly larger LA size and EROA, and more severe diastolic dysfunction (higher E/E’), regardless of symptom.

**Table 2:** Echocardiographic parameters of all patients with chronic organic mitral regurgitation and the comparisons between patients with and without pulmonary hypertension

Variables	Total (*n* = 154)	No PH (*n* = 113)	PH (*n* = 41)	*P*-value
RVSP (mmHg)	43.2 ± 17.7	34.6 ± 7.3	66.8 ± 16.1	<0.001
PAEDP (mmHg)	14.1 ± 6.5	11.6 ± 3.8	20.8 ± 7.6	<0.001
Mean PAP (mmHg)	25.8 ± 10.3	21.5 ± 6.9	36.8 ± 9.5	<0.001
PVR (Wood unit)	2.5(1.0–9.0)	2.2(1.0–25.2)	4.0(1.9–9.0)	<0.001
EROA (mm^2^)	61.8(22.0–250.9)	55.9(22.0–250.9)	87.5(27.0–225.1)	<0.001
RVol (ml)	107.1 ± 51.8	99.1 ± 48.4	129.1 ± 55.2	0.001
Severe MR	123(79.9)	85(75.2)	38(92.7)	0.017
LVEF (%)	68.1 ± 5.9	68.4 ± 5.9	67.2 ± 6.1	0.231
LAV (ml)	109.8(44.5–653.4)	94.3(44.5–486.3)	147.1(70.9–653.4)	<0.001
LAV index (ml/m^2^)	67.1(24.7–391.3)	59.3(24.7–276.3)	88.4(42.0–391.3)	<0.001
LA diameter (mm)	52.9 ± 11.1	50.0 ± 9.6	60.8 ± 11.1	<0.001
LVDd (mm)	56.6 ± 6.7	56.2 ± 6.5	58.8 ± 6.3	0.029
LVSd (mm)	33.8 ± 5.7	33.3 ± 5.9	35.2 ± 4.8	0.072
LVEDV (ml)	113.3 ± 33.0	110.2 ± 32.2	122.0 ± 33.7	0.049
LVESV (ml)	36.5 ± 13.6	35.0 ± 13.2	40.5 ± 13.9	0.027
LVMI (g/m^2^)	146.4 ± 43.4	139.4 ± 38.5	165.7 ± 50.7	0.001
E (cm/sec)	119.2 ± 33.8	108.7 ± 28.6	149.0 ± 29.8	<0.001
A (cm/sec)	70.8 ± 24.1	71.1 ± 24.7	69.3 ± 21.9	0.743
E/A	1.8 ± 0.8	1.6 ± 0.7	2.3 ± 1.0	<0.001
DT (ms)	201.9 ± 48.6	208.5 ± 50.5	182.7 ± 37.1	0.005
E/E’	15.9 ± 6.1	14.4 ± 5.3	20.7 ± 6.0	<0.001
TAPSE (mm)	22.7 ± 4.5	23.3 ± 4.3	21.2 ± 5.0	0.011
S_t_’ (cm/sec)	12.2 ± 2.2	12.3 ± 2.0	11.7 ± 2.7	0.148

Data are expressed as number (percentage), median (min-max) or mean ± standard deviation

*P*-values are for comparisons between 2 groups

*A* peak late diastolic velocity of mitral inflow, *DT*, deceleration time, *E* peak early diastolic velocity of mitral inflow, *E’* tissue Doppler peak early diastolic velocity of medial mitral annulus, *EROA* effective regurgitant orifice area, *LA* left atrium, *LAV* left atrial volume, *LV* left ventricle, *LVDd* left ventricular end-diastolic diameter, *LVEF* left ventricular ejection fraction, *LVSd* left ventricular end-systolic diameter, *LVEDV* left ventricular end-diastolic volume, *LVESV* left ventricular end-systolic volume, *LVMI* left ventricular mass index, *MR* mitral regurgitation, *PAEDP* pulmonary artery end-diastolic pressure, *PAP* pulmonary artery pressure, *PH* pulmonary hypertension, *PVR* pulmonary vascular resistance, *RVol* regurgitant volume, *RVSP* right ventricular systolic pressure, *S*_*t*_ *’* tissue Doppler peak systolic velocity of lateral tricuspid annulus, *TAPSE* tricuspid annular plane systolic excursion

**Table 3:** Baseline characteristics and echocardiographic parameters of patients with chronic organic mitral regurgitation and the comparisons between patients with and without pulmonary hypertension in the subgroups of patients with and without symptom

	Symptom (*n* = 58)			No symptom (*n* = 96)		
Variables	No PH	PH	*P*-value	No PH	PH	*P*- value
	(*n* = 34)	(*n* = 24)		(*n* = 79)	(*n* = 17)	
Age (years)	59.3 ± 15.7	56.9 ± 17.1	0.580	50.9 ± 16.6	62.6 ± 10.6	0.001
Male gender	18(52.9)	12(50.0)	0.825	51(64.6)	12(70.6)	0.635
Hypertension	20(58.8)	9(37.5)	0.110	23(29.1)	9(52.9)	0.059
Diabetes mellitus	4(11.8)	1(4.2)	0.392	4(5.1)	1(5.9)	1.0
Dyslipidemia	16(47.1)	5(20.8)	0.041	17(21.5)	6(35.3)	0.228
Smoking	13(38.2)	6(25.0)	0.290	23(29.1)	4(23.5)	0.772
PAEDP (mmHg)	12.6 ± 4.8	21.0 ± 7.5	<0.001	11.3 ± 3.3	20.4 ± 8.0	<0.001
EROA (mm^2^)	51.0(23–251)	98.4(36–207)	0.001	56.1(22–162)	70.5(27–225)	0.250
RVol (ml)	106.3 ± 63.1	138.6 ± 54.1	0.047	96.0 ± 40.5	115.5 ± 55.6	0.096
Severe MR	24(70.6)	23(95.8)	0.019	62(78.5)	15(88.2)	0.510
LVEF (%)	68.2 ± 6.43	65.9 ± 6.0	0.169	68.6 ± 5.6	69.0 ± 5.9	0.782
LAV (ml)	93.3(50.5–286.8)	156.3(86.4–653.4)	0.001	97.2(44.5–486.3)	133.4(70.9–197.9)	0.002
LA index (ml/m^2^)	57.4(30.3–178.9)	98.0(54.3–391.3)	0.001	60.1(24.7–276.3)	74.1(42.0–114.1)	0.004
LA diameter (mm)	52.6 ± 8.7	63.1 ± 12.5	<0.001	48.9 ± 9.9	57.6 ± 8.0	0.001
LVDd (mm)	55.9 ± 7.2	60.2 ± 7.3	0.029	56.3 ± 6.3	56.7 ± 3.9	0.713
LVSd (mm)	32.9 ± 6.8	36.8 ± 4.7	0.018	33.5 ± 5.6	32.9 ± 4.1	0.669
LVEDV (ml)	104.7 ± 35.3	127.6 ± 34.2	0.017	112.6 ± 30.8	114.1 ± 32.3	0.855
LVESV (ml)	33.6 ± 15.3	43.8 ± 13.8	0.012	35.7 ± 12.2	35.8 ± 13.0	0.959
LVMI (g/m^2^)	155.6 ± 44.8	175.4 ± 56.9	0.144	132.3 ± 33.2	151.1 ± 36.5	0.046
E/E’	16.2 ± 5.7	21.0 ± 5.8	0.006	13.6 ± 5.0	20.4 ± 6.5	<0.001
TAPSE (mm)	22.2 ± 3.9	20.5 ± 5.4	0.183	23.7 ± 4.3	22.2 ± 4.4	0.185
S_t_’ (cm/sec)	12.2 ± 2.2	11.4 ± 2.8	0.212	12.4 ± 1.9	12.1 ± 2.5	0.551

Data are expressed as number (percentage), median (min-max) or mean ± standard deviation

*P*-values are for comparisons between 2 groups

*A* peak late diastolic velocity of mitral inflow, *DT* deceleration time, *E* peak early diastolic velocity of mitral inflow, *E’* tissue Doppler peak early diastolic velocity of medial mitral annulus, *EROA* effective regurgitant orifice area, *LA* left atrium, *LAV* left atrial volume, *LV* left ventricle, *LVDd* left ventricular end-diastolic diameter, *LVEF* left ventricular ejection fraction, *LVSd* left ventricular end-systolic diameter, *LVEDV* left ventricular end-diastolic volume, *LVESV* left ventricular end-systolic volume, *LVMI* left ventricular mass index, *MR* mitral regurgitation, *PAEDP* pulmonary artery end-diastolic pressure, *PAP* pulmonary artery pressure, *PH* pulmonary hypertension, *PVR* pulmonary vascular resistance, *RVol* regurgitant volume, *RVSP* right ventricular systolic pressure, *S*_*t*_ *’* tissue Doppler peak systolic velocity of lateral tricuspid annulus, *TAPSE* tricuspid annular plane systolic excursion

### Determinants of pulmonary hypertension

**Fig. 1:**
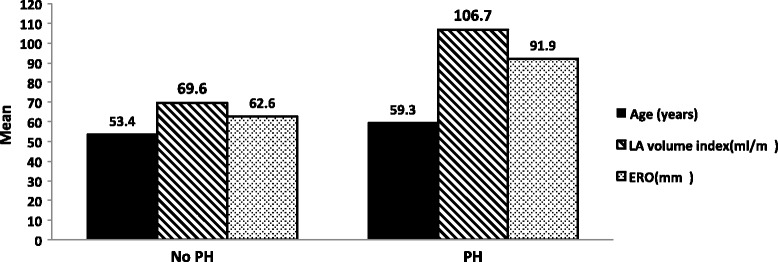
Independent determinants of pulmonary hypertension in patients with chronic organic mitral regurgitation (EROA = effective regurgitant orifice area; LA = left atrium; PH = pulmonary hypertension)

In univariate analysis, a number of factors were significantly associated with PH, including age, AF, LV mass index, LV end-systolic and end-diastolic volume, LAV index, TAPSE and EROA (Table 4). By multiple regression analysis, age (*p* = 0.044), LAV index (*p* = 0.037), and EROA (*p* = 0.006) remained independent predictors of PH (Table 4 and Fig. 1). According to ROC analysis to identify the optimal cut-off value of LAV index to predict PH, the area under the curve was 0.76. The sensitivity, specificity, predictive accuracy of positive and negative results at different values of LAV index are shown in Table 5.

**Table 4:** Univariate and multivariate factors associated with pulmonary hypertension

Factors	Crude odd ratio (95 % CI)	*P*-value	Adjusted odd ratio (95 % CI)	*P*-value
Age	1.02 (1.00–1.06)	0.053	1.03 (1.001–1.06)	0.044
AF	2.86 (1.34–6.08)	0.006	-	
LVMI	1.01 (1.01–1.02)	0.002	-	
LVEDV	1.01 (1.0–1.02)	0.052	-	
LVESV	1.03 (1.003–1.06)	0.031	-	
LAV index	1.02 (1.01–1.03)	0.001	1.01 (1.001–1.02)	0.037
TAPSE	0.90 (0.82–0.98)	0.013	-	
EROA	1.02 (1.01–1.03)	<0.001	1.02 (1.01–1.03)	0.006

*AF* atrial fibrillation, *CI* confidence interval, *EROA* effective regurgitant orifice area, *LAV* left atrial volume, *LVEDV* left ventricular end-diastolic volume, *LVESV* left ventricular end-systolic volume, *LVMI* left ventricular mass index, *TAPSE* tricuspid annular plane systolic excursion

**Table 5:** Cut-off value of left atrial volume index in predicting pulmonary hypertension

Cut off of LAV index	Sensitivity (%)	Specificity (%)	LR +	LR -	PPV (%)	NPV(%)
≥60 ml/m^2^	92.7 (80.1–98.5)	50.4 (40.9–60.0)	1.9 (1.5–2.3)	0.2 (0.1–0.4)	40.4 (30.4–51.0)	95.0 (86.1–99.0)
≥65 ml/m^2^	85.4 (70.8–94.4)	57.5 (47.9–66.8)	2.0 (1.6–2.6)	0.3 (0.1–0.5)	42.2 (31.4–53.5)	91.5 (82.5–96.8)
≥70 ml/m^2^	70.7 (54.5–83.9)	63.7 (54.1–72.6)	2.0 (1.4–2.7)	0.5 (0.3–0.8)	41.4 (29.8–53.8)	85.7 (76.4–92.4)

*LAV* left atrial volume, *LR* likelihood ratio, *NPV* negative predictive value, *PPV* positive predictive value

### Correlation between LAV index and pulmonary artery systolic pressure

There was a moderate to good positive correlation between LAV index and pulmonary artery systolic pressure (*r* = 0.51, *p* < 0.001). The correlation between LAV index and pulmonary artery systolic pressure remained moderate to good in the subgroup of asymptomatic patients (*r* = 0.59, *p* < 0.001), while the correlation was fair in symptomatic patients (*r* = 0.36, *p* < 0.001). Regarding the severity of MR, the correlations between LAV index and pulmonary artery systolic pressure was fair in the subgroup of patients with severe MR (*r* = 0.47, *p* < 0.001), and moderate to good in patients without severe MR (*r* = 0.54, *p* = 0.002). Table 6 showed the clinical and echocardiographic comparisons according to the symptom status and the severity of MR. Patients with severe MR or symptomatic patients were more likely to have diastolic dysfunction (higher E/E’ ratio), higher LV mass and LAV index and higher pulmonary artery systolic pressure than those without severe MR or asymptomatic patients. Regarding the magnitude of MR, there were fair positive correlations between LAV index and EROA (*r* = 0.47, *p* < 0.001), and regurgitant volume (*r* = 0.46, *p* < 0.001). Furthermore, patients with AF had a higher LAV index than those in sinus rhythm (median 98.1 (33.9–391.3) vs. 59.7 (24.7–202.4) ml/m^2^, *p* = 0.001).

**Table 6:** Clinical characteristics and echocardiographic parameters of patients with chronic organic mitral regurgitation and the comparisons between subgroups of patients according to the symptom status and the severity of mitral regurgitation

Variables	Total (*N* = 154)	Symptom status			Severity of MR		
		Symptomatic (*N* = 58)	Asymptomatic (*N* = 96)	*P*-value	Severe MR (*N* = 123)	Non-severe MR (*N* = 31)	*P*-value
Symptomatic	58 (37.7)	-	-	-	46 (37.4)	12 (38.7)	1.0
Severe MR	123(79.9)	46 (79.3)	77 (80.2)	1.0	-	-	-
Age (years)	55.0 ± 16.4	58.3 ± 16.2	53.0 ± 16.3	0.051	55.3 ± 16.1	53.9 ± 17.9	0.684
Male gender	93(60.4)	30(51.7)	63(65.6)	0.087	82(66.7)	11(35.5)	0.002
Atrial fibrillation	45(29.4)	25(43.1)	20(21.1)	0.004	34(27.9)	11(35.5)	0.406
LVDd (mm)	56.9 ± 6.6	57.7 ± 7.5	56.3 ± 5.9	0.245	58.3 ± 6.3	51.1 ± 4.1	<0.001
LVSd (mm)	33.8 ± 5.7	34.5 ± 6.3	33.4 ± 5.3	0.238	34.5 ± 5.8	31.0 ± 4.4	<0.001
LVEDV (ml)	113.3 ± 33.0	114.2 ± 36.4	112.8 ± 30.9	0.810	119.5 ± 32.3	89.0 ± 22.9	<0.001
LVESV (ml)	36.5 ± 13.6	37.8 ± 15.5	35.7 ± 12.3	0.382	38.0 ± 13.8	30.4 ± 10.5	0.005
LVMI (g/m^2^)	146.4 ± 43.44	163.8 ± 50.7	135.5 ± 34.3	<0.001	154.6 ± 42.5	113.3 ± 29.8	<0.001
E/E’	15.9 ± 6.1	18.0 ± 6.1	14.8 ± 5.9	0.002	16.6 ± 6.2	13.3 ± 5.0	0.008
TAPSE (mm)	22.7 ± 4.6	21.5 ± 4.6	23.4 ± 4.4	0.010	22.9 ± 4.5	22.0 ± 4.9	0.307
EROA (mm^2^)	61.8(22.0–250.9)	78.7(23.0–250.9)	56.5(22.0–225.1)	0.080	72.0(40.0–250.9)	28.9(22.0–44.9)	<0.001
RVol (ml)	102.1(20.3–336.2)	114.8(36.2–336.2)	95.5(20.3–267.9)	0.058	113.3(61.3–336.2)	48.1(20.3–75.0)	<0.001
LAV index (ml/m^2^)	67.1(24.7–391.3)	80.4(30.3–391.3)	62.5 (24.7–276.3)	0.007	71.7(30.3–391.3)	46.3(24.7–145.0)	<0.001
RVSP (mmHg)	43.2 ± 17.7	49.0 ± 18.0	39.6 ± 16.5	0.001	44.9 ± 18.7	36.3 ± 10.4	0.001

Data are expressed as number (percentage), median (min-max) or mean ± standard deviation

*P*-values are for comparisons between 2 groups

*E* peak early diastolic velocity of mitral inflow, *E’* tissue Doppler peak early diastolic velocity of medial mitral annulus, *EROA* effective regurgitant orifice area, *LAV* left atrial volume, *LVDd* left ventricular end-diastolic diameter, *LVSd* left ventricular end-systolic diameter, *LVEDV* left ventricular end-diastolic volume, *LVESV* left ventricular end-systolic volume, *LVMI* left ventricular mass index, *MR* mitral regurgitation, *RVol* regurgitant volume, *RVSP* right ventricular systolic pressure, *TAPSE* tricuspid annular plane systolic excursion

## Discussion

The present study emphasizes the importance of LAV index in the association with and as an independent determinant of PH in patients with chronic organic MR. The majority was due to chronic degenerative MR. PH was reported in 26.6 % of patients. Patients with PH had larger LAV than those without. Among clinical and echocardiographic parameters, age, AF, LV mass index, LV volume, LAV index, TAPSE and the severity of MR were univariately associated with PH. Importantly, age, LAV index, and the severity of MR as assessed by EROA remained independent determinants of PH.

### Left atrial enlargement and pulmonary hypertension in chronic organic mitral regurgitation

PH is a common and frequent complication in patients with chronic organic MR. A cutoff value of pulmonary artery systolic pressure >50 mmHg was employed in this study according to the current guidelines [11, 12]. Using this value, previous studies reported PH in 23–33 % of patients with organic MR [5, 9, 10]. Similarly, PH was observed in 26.6 % of patients in our study. PH as a common consequence of mitral valve disease is categorized as pulmonary venous hypertension [18]. The mechanisms of PH due to left-sided heart disease may be multiple and complex, but the common pathway leading to PH is presumably due to an elevated diastolic filling pressure of the left heart [18, 19]. In the setting of chronic elevation in the filling pressure, increased LA pressure and LA enlargement are expected. Several entities are known to result in an increased LA pressure and LA enlargement, including LA volume/pressure overload secondary to chronic mitral valve diseases, and elevated LV filling pressure from LV systolic/diastolic dysfunction. As a consequence of elevated LA pressure, there is a passive backward transmission of pressure to the pulmonary vascular bed, which triggers vasoconstriction in the pulmonary arterial bed, leading to PH [19].

In patients with chronic organic MR, LA enlargement and PH commonly co-exist. The regurgitant volume of MR adds an excess volume load to LA, leading to LA dilatation, an increased LA pressure and PH. The more severe the MR becomes, the more likely the LAV increases. Atrial fibrillation and PH eventually occur and lead to clinical symptoms and adverse clinical outcomes. The prognostic importance of LA enlargement and PH in patients with chronic organic MR has been well established [5–10]. Theoretically, LA enlargement and PH share the ultimate pathophysiological consequences of chronic organic MR. However, studies primarily verifying the link between LA enlargement and PH in patients with chronic organic MR are scarce. Ghoreishi M et al. reported that higher pulmonary systolic pressure was found in patients with a higher grade of MR, a larger LA dimension, and more right ventricular dysfunction [9]. Barbieri A et al. demonstrated similar findings that LA dimension was significantly higher in MR patients with PH as compared to those without [5]. Of note, LA dimension, not LAV, was used in those 2 studies. Le Tourneau T et al. studied 492 patients with chronic organic MR in sinus rhythm and the majority of patients (91 %) were asymptomatic [7]. The author found the relationship between the subgroups of LAV index (<40, 40 to 59, and ≥60 ml/m^2^) and pulmonary artery systolic pressure. However, recent study in asymptomatic patients with moderate or severe MR by Arias A et al. showed that there was no statistically significant difference in pulmonary artery systolic pressure between patient with LAV index <55 and ≥55 ml/m^2^ [8]. The present study confirmed the positive correlation between LA enlargement, using LAV, and PH in patients with MR, regardless of the presence of symptom or the severity of MR. However, the correlations between LA enlargement and pulmonary artery systolic pressure were fair in the subgroups of patients with symptoms or severe MR as compared to moderate to good correlations in the subgroups of patients without severe MR or asymptomatic patients. The pathophysiological consequences and morphological changes after MR are more pronounced in patients with symptom or severe MR than in those without (Table 6). Furthermore, the property of LA ifself, such as LA compliance, is the important determinant of subsequent LA size and the degree of pulmonary pressure during the progression of MR. These may explain the less robust correlations between LAV index and pulmonary artery systolic pressure in patients with more advanced disease.

### Determinant of pulmonary hypertension in mitral regurgitation

The present study is unique in demonstrating not only the association between LAV index and PH, but also the significance of LAV index as an independent determinant of PH in patients with chronic organic MR. Although there are several studies regarding the prognostic implication of LA enlargement and PH in patients with chronic organic MR, the issue verifying the importance of LA enlargement as an independent determinant of PH is under-recognized. Barbieri et al. demonstrated that age and LA size were independent predictors of PH in patients with MR [5]. However, their study used LA dimension, instead of LAV, and the study population was limited to those with degenerative MR due to flail leaflet. Furthermore, Le Tourneau T et al. emphasized the importance of LAV as an independent predictor of PH and PH as an independent predictor of post-operative adverse outcomes in patients with organic MR specifically referred for mitral valve surgery [20]. Our findings add more evidence supporting the association between LA enlargement and PH in any patient with chronic isolated organic MR, regardless of cardiac rhythm, the severity, the etiology and the timing for mitral valve surgery.

In the present study, apart from LAV index, age and the severity of MR as assessed by EROA were found to be the independent determinants of PH in patients with chronic organic MR. Previous studies in general population showed that age was associated with an increase in pulmonary artery systolic pressure as the result of diastolic dysfunction, an increased medial thickness and impaired elastic properties of pulmonary vessels [21, 22]. In patients with MR, the relationships between age, the severity of MR and PH have also been reported [5, 9]. Among three independent determinants of PH reported in the present study, it is essential to emphasize the significance of the severity of MR as assessed by EROA as the most powerful determinant. As expected from the pathophysiological viewpoint and the results of the outcome study, severe MR ultimately leads to more advanced hemodynamic alterations as evident by higher LAV index, presence of AF, marked LV dilatation and dysfunction, more severe PH, and impaired right ventricular function, and unfavorable outcomes [5, 6].

### Study limitations

The present study employed the proximal isovelocity surface area method as the only method to determine the severity of MR. Pulmonary artery pressure used in this study was determined solely by Doppler echocardiography, not by right heart catheterization. However, the estimations of MR severity and pulmonary artery pressure by Doppler echocardiography are reliably obtained and the current literature supports their values in routine clinical practice [13–16]. Nevertheless, in some difficult clinical settings, such as right heart failure or presence of primary tricuspid regurgitation, the echocardiographic estimation of pulmonary artery systolic pressure may be misleading. As often as is necessary for patient’s optimal therapeutic management, right heart catheterization with thermodilution method should be used to achieve a more reliable estimate of pulmonary artery systolic pressure. Although the relationship between LA enlargement and PH in patients with chronic organic MR is theoretically expected and has already been well established, it emphasizes the pathophysiologic viewpoint and may not provide substantial clinical impact. The generalization of the present study to all patients with chronic MR is limited and may be mainly applied to those with chronic degenerative MR as the majority of the study population. Furthermore, not only the severity, but also the duration of MR is a key determinant of PH and LAV in patients with MR. However, the exact duration of MR is difficult to obtain since the history taking itself may not be precise. Although the cut-off values of LAV index in predicting PH were proposed in the present study, it remains crucial to periodically assess the change of pulmonary artery pressure and perform the comprehensive echocardiographic examination during the follow-up in patients with chronic organic MR.

## Conclusion

In patients with chronic organic MR, a significant association exists between LAV index and PH. Age, the severity of MR as assessed by EROA and LAV index provide useful information as the independent determinants of PH. This finding supports the common pathophysiological and hemodynamic consequences of chronic organic MR.

## Abbreviations

A, peak late diastolic velocity of mitral inflow; A’, longitudinal late diastolic myocardial velocity; AF, atrial fibrillation; E, peak early diastolic velocity of mitral inflow; E’, longitudinal early diastolic myocardial velocity; EROA, effective regurgitant orifice area; LA, left atrial; LAV, left atrial volume; LV, left ventricular; MR, mitral regurgitation; PH, pulmonary hypertension; ROC, receiver operating characteristic; S_m_’, longitudinal systolic myocardial velocity of medial mitral annulus; S_t_’, longitudinal systolic myocardial velocity of lateral tricuspid annulus; TAPSE, tricuspid annular plane systolic excursion; TDI, tissue doppler imaging
